# Supplementary material for: Predictions of the Biological Effects of Several Acyclic Monoterpenes as Chemical Constituents of Essential Oils Extracted from Plants
Source: Molecules. 2023 Jun 8;28(12):4640. doi: 10.3390/molecules28124640 (PMC10302384; doi:10.3390/molecules28124640)
Supplement: Supplementary file 1 [file molecules-28-04640-s001.zip › molecules-2415534-supplementary.pdf]

Predictions of the Biological Effects of Several Acyclic Monoterpenes as Chemical Constituents of Essential Oils Extracted from Plants

Daniela Dascalu [1,2,†], Adriana Isvoran [1,2,†] and Nicoleta Ianovici [1,3,*]

[1] Department of Biology Chemistry, West University of Timisoara, 16 Pestalozzi, 300115 Timisoara, Romania; daniela.dascalu@e-uvt.ro (D.D.); adriana.isvoran@e-uvt.ro (A.I.)
[2] Advanced Environmental Research Laboratories, West University of Timisoara, 4 Oituz, 300086 Timisoara, Romania
[3] Environmental Biology and Biomonitoring Research Center, West University of Timisoara, 16 Pestalozzi, 300115 Timisoara, Romania
* Correspondence: nicoleta.ianovici@e-uvt.ro
† These authors contributed equally to this work.

Table S1. SMILES formulas and physicochemical properties of the acyclic monoterpenes considered in the present study.

Common name	SMILES formula	MW (g/mol)	logP	HBD	HBA	RB	tPSA (Å²)
beta-ocimene	CC(=CC/C=C(/C)\C=C)C	136.23	4.3	0	0	3	0.00
beta-myrcene	CC(=CCCC(=C)C=C)C	136.23	4.3	0	0	4	0.00
geranial (citral)	CC(=CCC/C(=C/C=O)/C)C	152.23	3.0	0	1	4	17.1
citronellal	CC(CCC=C(C)C)CC=O	154.25	3.0	0	1	5	17.1
geraniol	CC(=CCCC(=CCO)C)C	154.25	2.9	1	1	4	20.2
linalool	CC(=CCCC(C)(C=C)O)C	154.25	2.7	1	1	4	20.2
citronellol	CC(CCC=C(C)C)CCO	156.26	3.2	1	1	5	22.2
linalyl acetate	CC(=CCCC(C)(C=C)OC(=O)C)C	196.99	3.3	0	2	6	26.3
citronellyl acetate	CC(CCC=C(C)C)CCOC(=O)C	198.30	3.8	0	2	7	26.3

Table S2. ADMETlab2.0 outputs regarding the interactions of acyclic monoterpenes with human cytocromes strongly involved in metabolism of xenobiotics.

Compound/ activity	inhibitor					substrate				
	CYP1A2	CYP2C19	CYP2C9	CYP2D6	CYP3A4	CYP1A2	CYP2C19	CYP2C9	CYP2D6	CYP3A4
beta-ocimene	0.902	0.460	0.097	0.080	0.124	0.248	0.806	0.104	0.147	0.279
beta-myrcene	0.785	0.176	0.073	0.015	0.032	0.243	0.803	0.719	0.519	0.261
geranial (citral)	0.781	0.346	0.067	0.044	0.020	0.639	0.867	0.902	0.590	0.226
citronellal	0.578	0.116	0.037	0.049	0.033	0.406	0.669	0.728	0.269	0.188
geraniol	0.490	0.079	0.034	0.048	0.024	0.263	0.410	0.695	0.168	0.214

linalool	0.163	0.242	0.042	0.050	0.300	0.281	0.832	0.768	0.112	0.285
citronellol	0.650	0.092	0.052	0.007	0.018	0.427	0.604	0.765	0.115	0.162
linalyl acetate	0.258	0.161	0.038	0.091	0.398	0.161	0.842	0.196	0.113	0.314
citronellyl acetate	0.966	0.449	0.282	0.029	0.069	0.151	0.531	0.541	0.110	0.221

Table S3. Probabilities that the investigated acyclic monoterpenes to affect the nuclear receptors: AR—androgen receptor, ER—estrogen receptor, PPAR-gamma—peroxisome proliferator-activated receptor gamma.

Compound/ Effects on the nuclear receptors	AR	ER	PPAR-gamma
beta-ocimene	0.006	0.060	0.423
beta-myrcene	0.012	0.082	0.112
geranial (citral)	0.012	0.081	0.007
citronellal	0.005	0.140	0.004
geraniol	0.003	0.072	0.003
linalool	0.006	0.112	0.003
citronellol	0.006	0.114	0.003
linalyl acetate	0.012	0.074	0.005
citronellyl acetate	0.017	0.074	0.002

Table S4. Predictions Skin sensitization potential of the investigated acyclic monoterpenes: DPRA—direct peptide reactivity assay, KeratinoSens—a method using immortalised adherent cell line derived from human keratinocytes; H-CLAT—human Cell Line Activation Test; LLNA—Local Lymph Node Assay data; HRIPT/HMT—Human Repeated Insult Patch Test (HRIPT) and Human Maximization Test (HMT); AD—applicability domain of the model.

Compound	DPRA	KeratinoSens	h-CLAT	LLNA in vivo	HRIPT/HMT	Baesyan outcome
beta-ocimene	Sensitizer (Outside AD, Confiability 88.3%)	Sensitizer (Outside AD, Confiability 92.4%)	Sensitizer (Inside AD, Confiability 60.6%)	Non-Sensitizer (Outside AD, Confiability 97.9%)	Sensitizer (Outside AD, Confiability 96.2%)	Sensitizer
beta-myrcene	Sensitizer (Outside AD,	Sensitizer (Outside AD,	Sensitizer	Non-Sensitizer	Sensitizer	Sensitizer

	Confiability 92.6%)	Confiability 95.3%)	(Inside AD, Confiability 60.7%)	(Outside AD, Confiability 99.8%)	(Outside AD, Confiability 99.1%)	
geranial (citral)	Sensitizer (Inside AD, Confiability 95.1%)	Sensitizer (Inside AD, Confiability 99.2%)	Sensitizer (Inside AD, Confiability 60.6%)	Non-Sensitizer (Outside AD, Confiability 99.9%)	Sensitizer (Inside AD, Confiability 99.5%)	Sensitizer
citronellal	Sensitizer (Inside AD, Confiability 91.6%)	Sensitizer (Inside AD, Confiability 91.9%)	Sensitizer (Inside AD, Confiability 60.9%)	Sensitizer (Outside AD, Confiability 99.8%)	Sensitizer (Inside AD, Confiability 99.3%)	Sensitizer
geraniol	Non-Sensitizer (Inside AD, Confiability 86.6%)	Sensitizer (Intside AD, Confiability 97.5%)	Sensitizer (Inside AD, Confiability 58.9%)	Sensitizer (Outside AD, Confiability 99.9%)	Sensitizer (Inside AD, Confiability 78.5%)	Sensitizer
linalool	Non-Sensitizer (Inside AD, Confiability 90.1%)	Non-Sensitizer (Inside AD, Confiability 90.6%)	Sensitizer (Inside AD, Confiability 59.7%)	Non-Sensitizer (Outside AD, Confiability 98.5%)	Sensitizer (Inside AD, Confiability 99.6%)	Sensitizer
citronellol	Sensitizer (Inside AD, Confiability 90.1%)	Non-Sensitizer (Inside AD, Confiability 93.7%)	Sensitizer (Inside AD, Confiability 59.2%)	Non-Sensitizer (Outside AD, Confiability 100%)	Non-Sensitizer Inside AD, Confiability 93.1%)	Non-Sensitizer
citronellyl acetate	Sensitizer (Inside AD, Confiability 94.0%)	Non-Sensitizer (Inside AD, Confiability 56.1%)	Sensitizer (Inside AD, Confiability 60.0%)	Non-Sensitizer (Outside AD, Confiability 99.8%)	Non-Sensitizer (Inside AD, Confiability 80.8%)	Non-Sensitizer
linalyl acetate	Sensitizer (Inside AD, Confiability 91.4%)	Non-Sensitizer (Inside AD, Confiability 94.1%)	Sensitizer (Inside AD, Confiability 61.1%)	Sensitizer (Outside AD, Confiability 99.3%)	Sensitizer (Inside AD, Confiability 99.1%)	Sensitizer

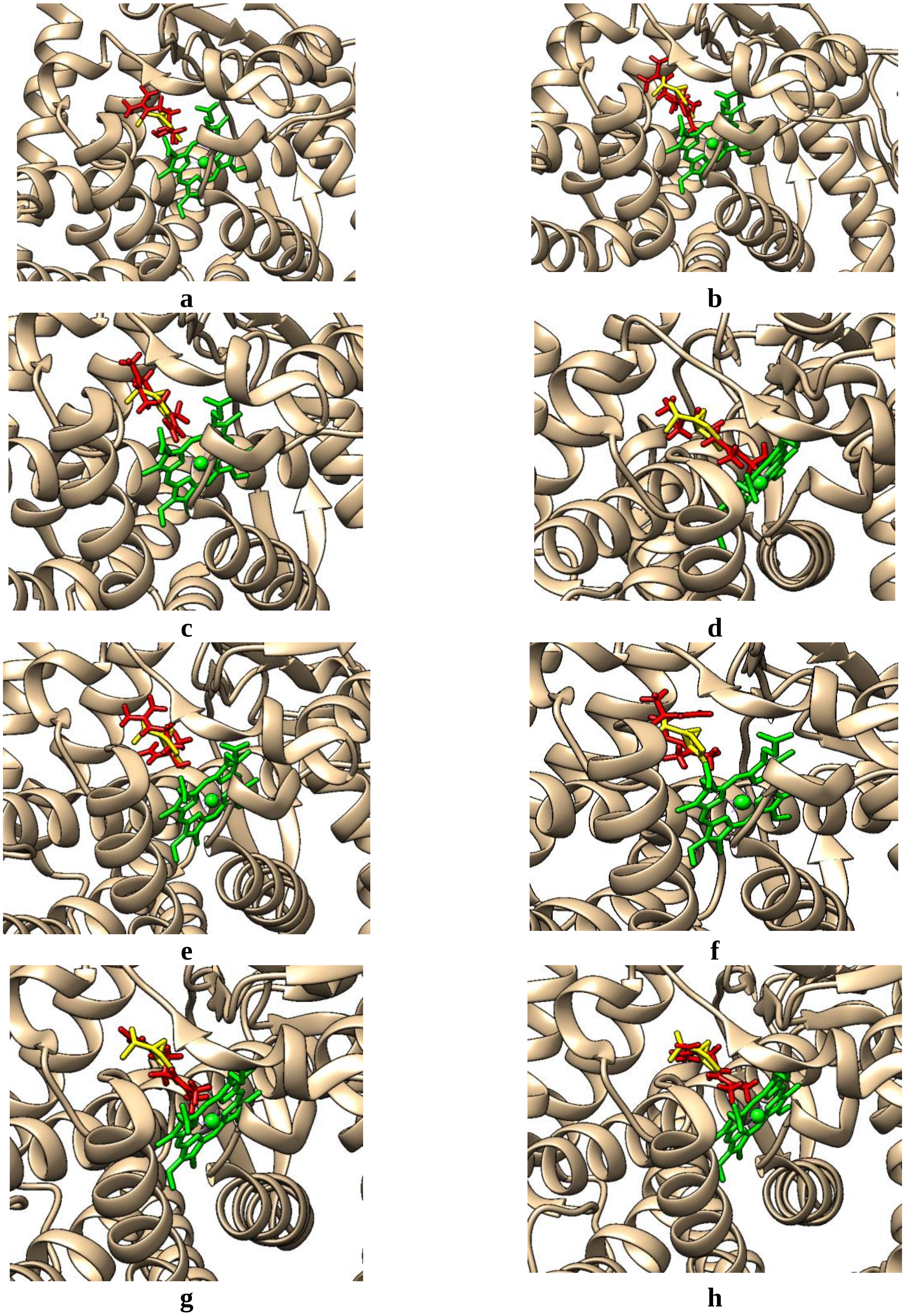

Figure S1. The binding poses of the acyclic monoterpenes to human CYP2B6: (**a**) citronellol, (**b**) citronellyl acetate, (**c**) geranial, (**d**) geraniol, (**e**) linalool, (**f**) linalyl acetate, (**g**) beta-myrcene, (**h**) beta-ocimene. The enzyme is revealed as brown ribbon, the acyclic monoterpenes are presented in red sticks, sabinene (the terpene that is found in the crystallographic structure of the enzyme) is revealed in yelow sticks and the prosthetic group hem in presented in green sticks.
